# Evaluating local primary health care actions to address health inequities: analysis of Australia’s Primary Health Networks

**DOI:** 10.1186/s12939-023-02053-8

**Published:** 2023-11-21

**Authors:** Alice Windle, Sara Javanparast, Toby Freeman, Fran Baum

**Affiliations:** 1 College of Nursing and Health Sciences, Flinders University, Adelaide, South Australia; 2 Stretton Health Equity, School of Social Sciences, Faculty of Arts, Business, Law and Economics, The University of Adelaide, Adelaide, South Australia

**Keywords:** Primary health care, Primary care, Health equity, Primary Health Network, Social determinants of health

## Abstract

**Background:**

Meso-level, regional primary health care organisations such as Australia’s Primary Health Networks (PHNs) are well placed to address health inequities through comprehensive primary health care approaches. This study aimed to examine the equity actions of PHNs and identify factors that hinder or enable the equity-orientation of PHNs’ activities.

**Methods:**

Analysis of all 31 PHNs’ public planning documents. Case studies with a sample of five PHNs, drawing on 29 original interviews with key stakeholders, secondary analysis of 38 prior interviews, and analysis of 30 internal planning guidance documents. This study employed an existing framework to examine equity actions.

**Results:**

PHNs displayed clear intentions and goals for health equity and collected considerable evidence of health inequities. However, their planned activities were largely restricted to individualistic clinical and behavioural approaches, with little to facilitate access to other health and social services, or act on the broader social determinants of health. PHNs’ equity-oriented planning was enabled by organisational values for equity, evidence of local health inequities, and engagement with local stakeholders. Equity-oriented planning was hindered by federal government constraints and lack of equity-oriented prompts in the planning process.

**Conclusions:**

PHNs’ equity actions were limited. To optimise regional planning for health equity, primary health care organisations need autonomy and scope to act on the ‘upstream’ factors that contribute to local health issues. They also need sufficient time and resources for robust, systematic planning processes that incorporate mechanisms such as procedure guides and tools/templates, to capitalise on their local evidence to address health inequities. Organisations should engage meaningfully with local communities and service providers, to ensure approaches are equity sensitive and appropriately targeted.

## Introduction

### Primary health care organisations - local action for health equity

Health inequity is unfair and unjust. Equity in health is defined as “the absence of disparities in health that are systematically associated with social advantage/disadvantage” [1]. Where health inequity can be avoided by reasonable action, the sustained existence of this inequity is unfair, and there is an ethical obligation to reduce it [2]. Inequity and inequality have a detrimental effect on societies – more equitable societies experience better outcomes on a wide range of health and social measures [3]. Health inequity is a consequence of “a combination of poor social policies and programmes, unfair economic arrangements, and bad politics” which lead to the “unequal distribution of power, income, goods, and services” (2, p1661). These factors shape the social determinants of health - the conditions of daily life in which people “are born, grow, live, work and age” (2, p1661). These include education, employment, income, housing, social inclusion, food security and more [2]. They are affected by the broader socioeconomic and political context and interact with individual factors (intermediary determinants) and the health care system itself, to influence the distribution of health in populations. A social gradient in health exists within and between countries where people with a lower socioeconomic status experience higher levels of illness and premature mortality [2]. The lower the socioeconomic level, the poorer the health status and outcomes. For example, internationally, life expectancy increases across the social gradient, with low income countries experiencing lower life expectancy than medium and high income countries. Infant mortality rates decline, the greater the income of the country [4]. Within Australia, there are clear correlations between socioeconomic disadvantage and poor health, on various indicators such as premature mortality and chronic disease prevalence, among others [5].

Comprehensive primary health care (PHC) strives for the goal of equitable health for all, through health promotion, disease prevention, community development, and intersectoral action on the underlying social determinants of health, as well as universally accessible primary care clinical treatment [6]. Cross- and intra-national comparisons have shown that strong PHC is associated with more equitable distribution of health across populations [7] and improved population health [8]. Comprehensive PHC can mitigate health inequity through equitable access to healthcare services, and action on the social determinants of health [6, 9]. As well as acting on ‘downstream’, individual factors by treating illness, comprehensive PHC employs ‘midstream’ and ‘upstream’ systemic interventions to address the underlying ‘causes of the causes’ of health inequity.

Meso-level primary health care organisations (PHCOs) are decentralised regional/local planning bodies for PHC, and feature in the health systems of several high-income countries. Being locally based organisations, they are well placed to improve population health through planning and development of PHC and population health interventions, being responsive to the needs of local communities. PHCOs can co-ordinate and integrate individual, organisational or local system activities at the juncture of primary care and public/population health - a well-recognized need [10–12]. Such integration has many benefits: improved chronic disease management; communicable disease control; better access to, and quality of care; greater efficiency; enhanced patient satisfaction; and better coordination and continuity of care [13].

In Australia, Primary Health Networks (PHNs) perform these meso-level functions and are funded by the federal government. They are responsible for assessing local population health needs, engaging with local communities and health system stakeholders, and planning PHC strategies to address priority issues. Rather than delivering services themselves, PHNs commission other organisations to deliver services and population health programs. A change of government saw thirty-one PHNs established in 2015 to replace the previous 61 Medicare Locals (MLs) which also performed this meso-level role. This followed a scathing but widely criticised review process, and claims of MLs’ activities adding an unnecessary layer of bureaucracy [14].

PHNs are well placed to drive local system change for more integrated care [15] and have the opportunity to act at both individual and population levels to integrate PHC with public health [16]. However, PHNs risk focussing too narrowly on clinical services, rather than acting further upstream. These concerns were identified early amidst governments’ rhetoric emphasising frontline services, individual *patients* and clinical *medical* services, while de-funding population health initiatives [16].

### A framework for primary health care organisations’ equity action

**Fig. 1:**
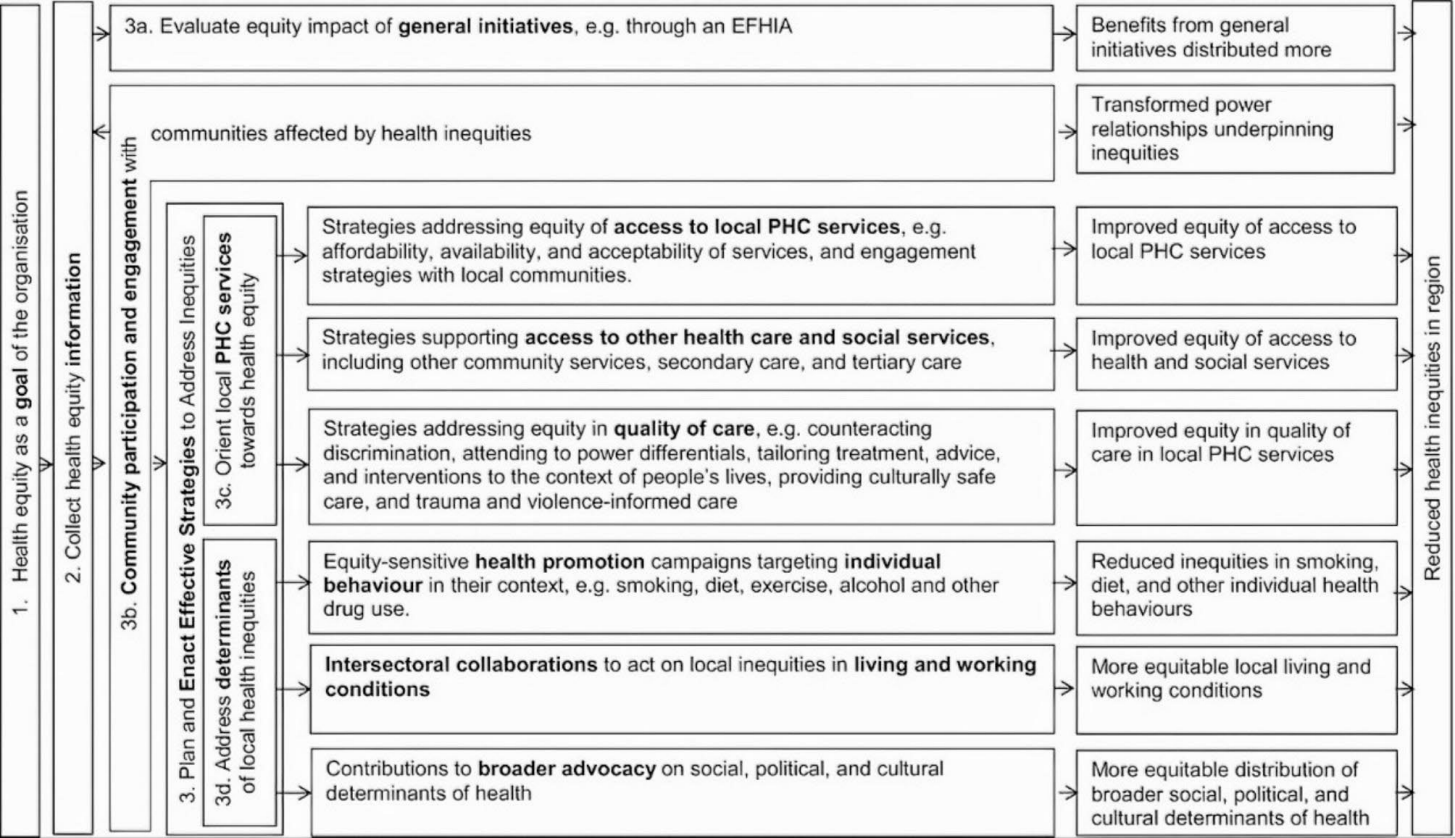
Framework for assessing regional primary health-care organisations actions on health equity (Freeman, et al., 2018). (Reproduced with permission of the copyright holder)

Freeman et al. [17] developed a framework to critically examine PHCOs’ actions on health equity, aligned to comprehensive PHC (Fig. 1). This is the only such framework of its type. It was developed based on theory and literature relating to health equity goals and strategies to address inequity in PHC, and was designed to measure PHC organisations’ health equity efforts against the scope of actions available, and allow for international comparison of such organisations. It was previously used to assess the equity actions of MLs - PHNs’ predecessors.

### Aims

This research aimed to:

- assess the equity actions of PHNs by applying the framework;
- identify factors that influence the equity-orientation of PHNs’ planned activities;
- contribute to evaluating the utility of the equity framework through a repeated application.

## Methods

We applied the framework to examine PHNs’ actions to address health inequities, and influences on equity-oriented planning. We gathered data through the following sources:

1. PHNs’ planning documents. We collected needs assessments, activity work plans (core, flexible funding) and annual reports from websites of all 31 PHNs. We examined: the inclusion of equity as a goal, objective or strategy; mentions of health equity issues and data; and indications of equity-oriented activities.
2. Interviews with PHN stakeholders. Five PHNs were selected as case study sites, including urban and rural/remote PHNs in various states/territories. (For anonymity, referred to as Metro North, Metro South^1^, Rural North, Rural South, and Remote.) PHNs were selected to capture a diverse range of states, rurality [18] and degrees of cultural diversity and socioeconomic conditions, and together they are broadly representative of PHNs nationally. Four of these had participated in an earlier phase of the research in 2016, which involved interviews with PHN executives, staff and members of boards, clinical councils and community advisory committees (see [19]). The research team undertook secondary analysis on these interview transcripts (n = 38) [20].
  Twenty-nine further semi-structured interviews were conducted in 2018 with equivalent stakeholders from the five case study PHNs. Interviews were conducted face-to-face at the PHN, or via telephone, according to a guide developed to examine PHC planning influences, capacity and processes, as they relate to equity considerations. Interviews were conducted in private and ranged in duration from approximately 60 to 80 min. Interviews took place between May and September 2018, and none were repeated. Two pilot interviews were conducted with people from non-participating PHNs. Interviews were conducted by the first author, a PhD candidate with experience in qualitative interviewing, and in PHCO planning roles. Two of the interviewees had prior professional peer interactions with the interviewer, and the rest had only a preliminary introduction to the research and interviewer prior to participation. Table 1 shows the number and mode of interviews, by PHN. All interviews were digitally recorded and professionally transcribed. All interviewees were offered the opportunity to review their transcript prior to analysis, but none did.
3. Case study document analysis. We conducted a focussed analysis on activity work plans of the five case study PHNs to examine their planned activities and their equity-orientation. We also analysed their internal documents relating to the process and /or principles for PHC planning. Four of the PHNs provided 26 documents. One PHN did not provide any internal documents, despite repeated requests. Four documents were sourced from their website, which were similar to those provided by other PHNs, or were the populated versions of templates.

**Table 1:** Primary Health Network interview participant numbers

PHN (codename)	Number of 2016 interviews	Number of 2018 interviews
Metro North	10	6
Metro South	7	6
Rural South	11	6
Remote	10	6
Rural North	Not applicable	5

Document and interview analysis was conducted using NVivo software [21]. A deductive coding approach was employed, using a coding framework developed by the research team to address the research questions and informed by the PHCO equity actions framework [17]. The part of the coding framework relating to equity issues sub-categories was developed in line with those used in the original primary analysis of the 2016 interviews. The coding framework was tested with one of each type of document and some minor modifications made.

Ethics approval was granted by the Flinders University Research Ethics Committee (#6376).

## Results

This section uses the framework [17] to first examine PHN intentions for addressing health inequity, then looks at which equity issues PHNs focused on. We then examine PHNs’ actions to address health inequity, and factors that influence equity-oriented planning.

### Equity intentions and conceptions

Intentions to improve equity were common in PHNs’ planning documents − 22 of the 31 PHNs (71%) stated goals for addressing health inequity in their region. There were also hundreds of statements of more specific equity-oriented objectives and strategies in 26 of the 31 PHNs’ planning documents (84%).

Intentions to reduce health inequities were also frequent in PHN interviews:

*“the board is not involved in saying, “commission organisation X to do Y”, we’re involved in saying, “We want to make a difference in inequalities in these areas and your job now is to go away and design services and commission services to address that”” (Board, Metro South, 2018)*.

In contrast to widespread good intentions, some PHN documents showed limited understanding of the complex, systemic relationships between socio-economic disadvantage and health, instead ascribing poor health to deficits in individuals’ attitudes, knowledge and skills, or unhealthy ‘choices’, frequently framed using the term ‘health literacy’.

*“The aim of this activity is to address health disparities through improved access to information, resources and skills … Improved health literacy enables people to make informed choices in regards to their health and supports the application of skills and knowledge to act on understanding.” (activity work plan)*.

In several PHNs, behavioural strategies were framed as aiming to “*activate*” or “*mobilise*” patients to change their behaviour, implying that patients were seen as unmotivated.

### Collection of health equity information

**Fig. 2:**
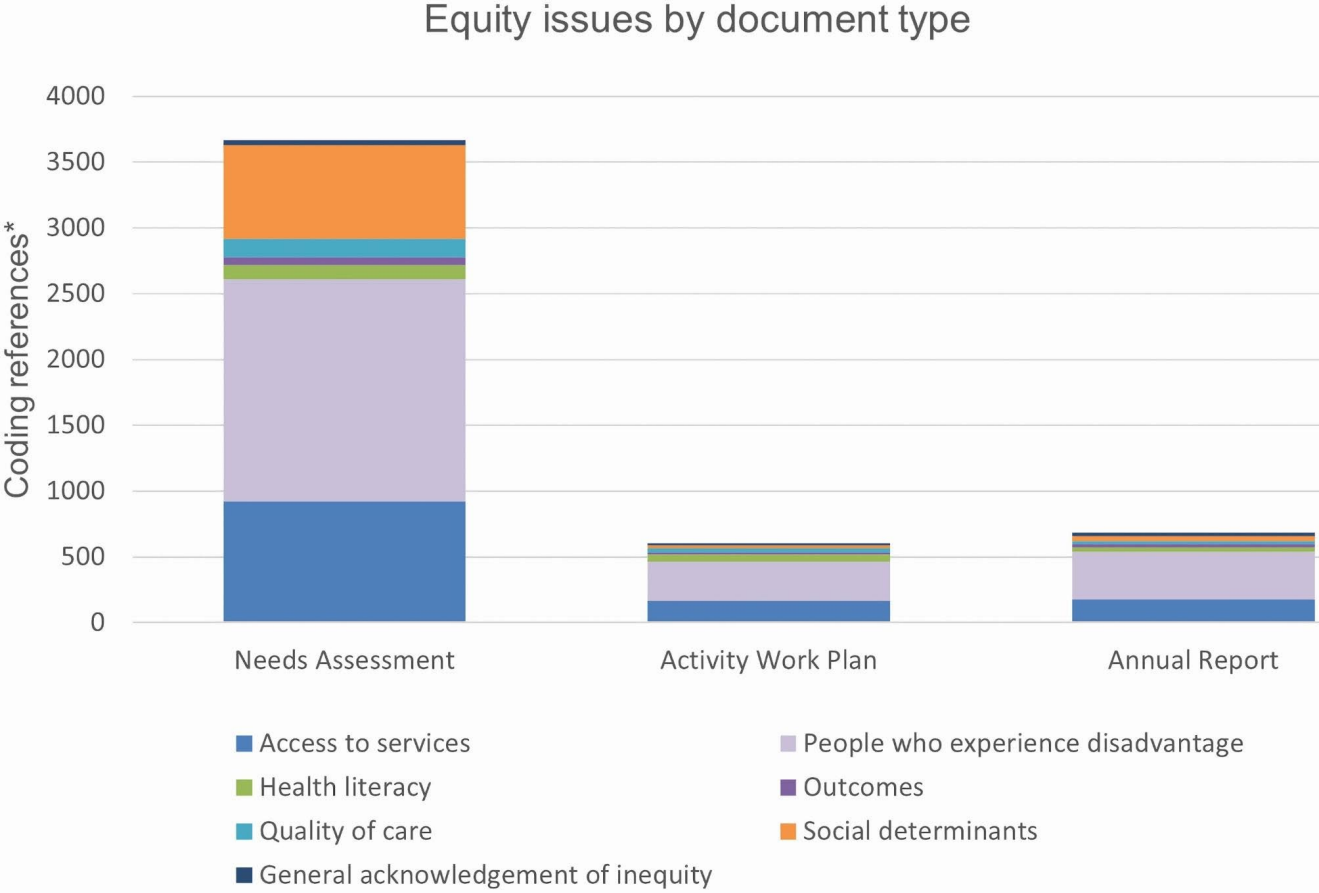
Equity issues in Primary Health Network documents, by document type (count). *Coding references are sections of text that refer to, and are coded to, one of the listed equity issues/codes. The count of coding references indicates the frequency with which the issue is discussed in documents but does not reflect the number of words dedicated to the equity issue in question

We found that PHNs collate evidence on health inequities to varying extents. Equity issues were much more frequently mentioned in needs assessments than activity work plans and annual reports (where information about funded activities is presented) (Fig. 2), suggesting that identification of equity issues is more common than action.

Figure 2 also illustrates the prominence of certain equity issues within PHN public documents. PHNs’ primary focus regarding inequity concerns people who experience disadvantage - predominantly First Nations people and people from culturally and linguistically diverse communities. Access to clinical services was a somewhat prominent equity issue in all document types.

There was greater focus on social determinants of health in needs assessments than in the other two types of documents. This suggests that while there is much acknowledgement and examination of the social determinants of health in identifying population health needs, there is little action on these.

Other equity themes such as quality of care, health outcomes, health literacy and general acknowledgement of inequity were sporadically discussed in the documents.

### Primary Health Network activities to address inequity

We analysed PHN activity work plans using the PHCO equity actions framework [17]. Table 2 shows our focussed analysis of the five case study PHNs, identifying a range of actions that can address inequity.

**Table 2:** Counts of case study Primary Health Network planned activities that are equity-oriented

Equity-oriented activities	Plan and enact effective strategies to addressinequities: orient local PHC services towards health equity				Plan and enact effective strategies to address inequities: address determinants of local health inequities			**Activities not evidently equity-oriented**
	**Strategies addressing equity of **access to local PHC services	**Strategies addressing equity in **quality of care	**Strategies addressing equity in** access to and quality of **PHC services**	**Strategies supporting** access to other health care and social services	**Equity-sensitive** health promotion **targeting** individual behaviour	Intersectoral collaborations **to act on local inequities in** living and working conditions	**Contributions to** broader advocacy **on social, political, and cultural determinants of health**	
Metro North	3	1	2	1	?			8
Metro South	2		1					11
Rural North	3		1		?			5
Remote	2		1					5
Rural South	4	3	1		?			6
Examples	Outreach allied health care to vulnerable children (Metro North)	Community of practice and provider support to integrate care of heart failure patients in identified areas (Rural South)	Care coordination services for people with complex needs, including dedicated component for Aboriginal and Torres Strait Islander people (Rural North)	PHC and other social support services for Aboriginal and Torres Strait Islander people (Metro North)	(*Behavioural interventions identified, but insufficient detail to determine if equity-sensitive)*			HealthPathways – information for clinicians on local health referral pathways (all PHNs, although Remote PHN outlined special consideration for health needs of Aboriginal and Torres Strait Islander people)
	PHC services in rural/remote areas (Remote, Rural North, Rural South)							
					Weight management and lifestyle education delivered via general practice in selected locations with high obesity rates (Rural North)			
								Provider support in digital health initiatives (Rural South, Rural North, Remote)
			Cross sectoral collaboration addressing child health needs in sub-region with high rates of social disadvantage (Metro North)					
	Immunisation for newly arrived refugees							
					Quality use of medicines initiative, in selected locations, targeting providers and community (Rural South)			

#### Actions to orient local primary health care services towards health equity

The most common approaches planned by the five case study PHNs were associated with improving access to clinical services and quality of care, although clearly stated objectives to distinguish between access and/or quality aims were infrequent.

Few activities aimed to facilitate access to other health and social services. An exception was one PHN’s services for refugees that included general practice and linkage with other social services.

#### Health promotion targeting individual behaviour

Behaviour change strategies were moderately common in PHN activity work plans overall. Some such strategies overlapped with the objective of increasing access to PHC services, for example an information campaign to promote PHC service options as alternatives to hospital presentation.

Around half of the behavioural health promotion strategies identified in PHNs appeared to have a clear equity objective, in that they focussed on particular community groups or subregions. Other than such targeting, it was generally not possible to determine whether the intervention was equity-sensitive.

As discussed above, behaviour change strategies were frequently framed as addressing individuals’ knowledge, behaviour and attitude deficits. Only a couple recognised an underlying reason for the need. For example, bilingual community educators to provide health information to refugees.

#### ‘Upstream’ health promotion and social determinants of health actions

Our analysis found very few activities that involved ‘upstream’, intersectoral action on living and working conditions to address the causes of health inequity. The few examples tended to include PHN participation in a broad network of multi-sectoral stakeholders, or activities with a narrow focus and not necessarily equity-oriented. For example: working with local government or transport agencies to promote active transport; or working with Alzheimer’s Australia to implement ‘dementia friendly environments’. Only one activity approximated broader advocacy, although it was focussed on influencing national primary care policy rather than upstream determinants of health.

Our analysis has shown that the strategies and actions planned by PHNs focus on individualistic clinical services and behaviour change initiatives, and fall short of employing a comprehensive PHC approach that would address health inequity more effectively.

### Influences on equity-orientation in primary health care planning

Through our PHN interviews we identified numerous factors that enable or hinder equity orientation in planning. These include:

#### Stakeholder engagement

Numerous interviewees in all five case study PHNs recognized that connection with relevant actors greatly enabled equity-oriented planning, drawing on their experience and knowledge to inform planning decisions and program design. This could take many different forms, including: robust and respectful community engagement relationships; representatives from communities experiencing disadvantage on advisory committees; connection with service providers with clinical experience in communities experiencing disadvantage; and board members from First Nations healthcare peak bodies.

The inclusion of representatives from communities experiencing disadvantage on community advisory committees, program steering groups or co-design committees was seen to help decision-makers to understand the complexity of issues.

*“… because we have that very strong community representation within the PHN structure. So you are hearing about that first hand often a lot more than perhaps may have existed … ensuring that it is well-informed by the right people [from the target population groups]” (Senior Executive, Rural North, 2018)*.

As well as bringing ‘lived experience’, it was identified by one interviewee (Rural South) that such representatives were expected to ask critical questions about equity related considerations of the programs being put forward. This mechanism for considering equity in planning is not particularly systematic and relies on people asking the right questions at the right time, and having an understanding of the drivers of inequities.

Achieving appropriate representation was identified as challenging by two PHNs. One had experienced significant difficulty in recruiting First Nations people to the community advisory committee, and also felt that the LGBTIQA + community was not represented. An interviewee from another PHN expressed concern at a lack of connection with people who experience disadvantage and lack of diversity on the community advisory committee:

*“I think that [committee establishment/ recruitment] process was a little bit flawed. The same sort of thing that we got previously with articulate middle-class people and doing a really good job in terms of advocating for their particular community” (Board, Rural North, 2018)*.

#### Planning process mechanisms to consider equity

While there were strong indications of organisational culture and leadership which supported equity-oriented planning, our analysis showed that systematic mechanisms to consider equity in planning (such as criteria) were rare. Instead, consideration of equity was frequently framed as a ‘lens’ through which decisions were considered.

Of the 30 internal documents analysed, 11 made no reference to equity considerations and nine included general rhetoric about equity, such as reiterating organisational goals or values. Only one PHN had clearly documented prompts for equity consideration in program planning, in relation to considering the impact of a program on First Nations people. A few documents included mechanisms for guiding commissioning decisions (such as preferencing Aboriginal Community Controlled Health Organisations for First Nations peoples’ health services), rather than planning and design decisions.

#### Other influences on equity-oriented planning

The strongest influence which hinders equity-orientation, is the tight constraint imposed by the federal government, which narrows the scope of activity that PHNs can plan.

*“we have the ability to identify gaps in equity, but we have a very limited ability to address them.” (Board, Remote, 2016)*.

Many interviewees noted that these limitations reflected the individualistic ideology of the incumbent government together with a selective, clinically-focussed interpretation of PHC. PHNs’ limited autonomy was seen to contrast with the idea of “locally relevant” planning and decision-making espoused by the Department of Health [22].

The tight timeframes imposed on PHNs were also seen to hinder their ability to conduct robust evidence-informed, equity-oriented planning, and ensure culturally safe practices:

*“the timelines that PHNs are being asked to respond are utterly ridiculous. Utterly, utterly ridiculous and in some ways, I would consider it disrespectful. Disrespectful to the organisations and but also the community.” (Staff, Remote, 2016)*.

In contrast, many interviewees stressed the enabling force of values, leadership and organisational culture endorsing health equity, which was evident in all five case study PHNs.

*“[Equity] is a very very strongly held value and really affects a lot of the conversations that we have at the board level” (Board, Remote, 2018)*.

There were clear indications from all PHNs of altruistic intentions and desire to “*make a difference*” (11 interviewees) indicating a strong equity culture within PHNs. There was however evidence of tension between the equity-conscious ideology of PHNs, and the priorities of federal or state/territory governments.

The need for good evidence in enabling equity-oriented planning was a strong theme in interviews. The tangible benefits of analysing and using high quality data to identify variation between different population groups were cited frequently.

*“by drilling down, you identify areas where there’s high need… you’ve got evidence of the need to continually work in that area.” (Board, Rural North, 2018)*.

*“a thorough understanding of our needs allows us to better meet the needs of our community.” (Clinical Council, Metro North, 2018)*.

The ability to use an evidence-informed population health approach and commissioning levers to help address the inequitable maldistribution of health services was particularly noted.

*“the mapping of our whole region shows very clearly hotspots where we have greatest need for mental health services, and we have the least services available, and where we have the worst socio-economic situations. When we map our [commissioned] services now they absolutely mirror those same areas. So we are delivering and causing those services to be directed more towards those areas of need and less just dictated by where the GPs are. Because in the past a lot of those services were going to the leafy green affluent suburbs, because that’s where the providers were; and now we’re getting that more directed to [disadvantaged] areas … we have capped services in the more advantaged areas, and left it uncapped in the disadvantaged areas and, after quite a few years, we see that complete change in how that program is delivered and the outcomes that it’s achieving, which is great … we knew that there was a lot of cherry picking going on when the market was left to its own devices” (Senior Executive, Metro North, 2016)*.

## Discussion

In the discussion we firstly compare our findings with those reported in the original use of the equity framework to determine how well it allows comparisons. We then explore explanations for PHNs’ limited equity actions and international examples of upstream equity action and systematic planning processes that incorporate mechanisms to embed equity considerations in planning.

### Use of the framework to compare equity-orientation of primary health care organisations

Table 3 outlines a summary of findings regarding PHNs and MLs’ actions on health inequity, and shows that generally PHNs and MLs were very similar. While it is disappointing that there doesn’t appear to have been much progress in PHC health equity action, it is reassuring that it doesn’t appear to have got significantly worse, particularly in the context of the neoliberal, individualistic rhetoric that surrounded the replacement of MLs with PHNs by the incoming government [16]. This may reflect the strong organisational culture and values driving equity that we observed within PHNs. Nonetheless, the reorganisation of MLs to PHNs represents a missed opportunity to incorporate mechanisms to promote health equity through local PHC action.

**Table 3:** Summary of Primary Healthy Networks’ actions on health equity

Health inequity action (from framework by Freeman et al. [17])		Extent of action by PHNs	PHN examples, evidence basis	Extent of action by MLs [17]
Health equity as a **goal** of the organisation		High	71% of PHNs state clear goals for addressing health inequity	High
Collect health equity **information**		Varies	Presence of equity issues in needs assessments varied between PHNs. Some very high.	High
Evaluate equity impact of **general initiatives**		Low	Very few indications of systematic process mechanisms to consider equity impacts of planned strategies	Low
**Community participation and engagement** with communities affected by health inequities		Moderate	All PHNs have a Community Advisory Committee and include community engagement as part of needs assessment and planning, but quality of engagement varies. (Inclusion of people from communities affected by inequities was not systematically assessed, but was evident in some PHNs)	Moderate
Plan and **enact effective strategies** to address inequities: orient local **PHC services** towards health equity:	Strategies addressing equity of **access to local PHC services**	High	E.g. on-site allied health services at selected schools, for children who have been identified as developmentally vulnerable and have experienced trauma E.g. funding general practice nurses to maintain recall and reminder systems, to support immunisation, targeting regions or population groups with lower immunisation rates	High
	Strategies supporting **access to other health care and social services**	Low	E.g. Education, training and resources for primary care providers on appropriate referral pathways for domestic violence	Low
	Strategies addressing equity in **quality of care**	Moderate	E.g. facilitating workforce capacity building to support the delivery of culturally appropriate care	Low
Plan and **enact effective strategies** to address inequities: address **determinants** of local health inequities	Equity-sensitive **health promotion** campaigns targeting **individual behaviour**	Moderate	E.g. wellness program for Aboriginal and Torres Strait islander people on nutrition, diabetes and child health E.g. community health literacy program for refugees	Moderate
	**Intersectoral collaborations** to act on local inequities in **living and working conditions**	Very low	E.g. participate in a regional child and youth mental health plan addressing (among other things) child protection/family violence E.g. Work with local council to encourage active recreation and active travel	Low
	Contributions to **broader advocacy** on social, political, and cultural determinants of health	None		None

### Primary Health Networks’ limited equity actions - constrained scope and victim blaming ideology

Our analysis highlighted the tendency for health equity strategies to be stated in planning documents but then drift to lifestyle interventions [23]. Most PHNs’ planning documents included intentions to address health inequities and presented considerable evidence to identify local health inequities, including social determinants. However, the activities planned by PHNs largely entailed ‘downstream’ clinical service-based interventions, and some individual behaviour change interventions, with negligible ‘midstream’ or ‘upstream’ action to address the social determinants of health equity. One of the key drivers for this disjuncture is that PHNs’ actions for health equity were required to align with the federal government’s narrow conception of health as an individual, biomedical concept, to be addressed by medical care or individual behaviour change [19]. The interests and power of the medical sector driving selective PHC ideas, and neoliberal ideas of economic imperatives and market models have been recognised as underlying factors that have hindered the pursuit of comprehensive, equity-oriented PHC in Australian PHCOs [24].

A recent UK study of local authority policy development for equity in children and young people [25] similarly found that there was a disconnect between national and local policy development and implementation, which allowed little flexibility for using local knowledge. They similarly identified a failure of national policy to take account of regional disparities and underlying social determinants of health inequity through ‘upstream’ action, which hindered local efforts to address inequity. As with our analysis of PHNs, Holding et al [25] identified some limitations and inconsistencies among key stakeholders’ understandings and definitions of inequalities, despite a widespread desire to address them.

PHNs’ equity-oriented culture and values, and considerable effort in identifying local priority needs and inequities is undermined by their limited ability to act. A similar tendency towards such ‘lifestyle drift’ has been noted in other settings [26, 27], where it contributes to health equity policy failure, alongside (and arguably reflecting) a fundamental inability to integrate policy and services, and a lack of political will for the necessary shift that disrupts the dominant biomedical, individualist hegemony [26]. Individualistic interventions are more politically palatable with neoliberal governments [28], but can exacerbate inequities [27, 29], so any ‘drift’ towards such approaches away from upstream approaches will compromise equity impact. While challenging to carry out, ‘bottom up’, participatory approaches are recognised as helping to stem ‘lifestyle drift’ [27]. Our study has similarly found that strong community and stakeholder engagement enabled equity-oriented PHC planning. Adequate resourcing for community participation in program development and implementation, combined with greater scope to enact upstream primary prevention strategies, acting locally on the underlying causes of ill-health would enable PHNs to have greater impact on health inequities.

Most PHNs stated health equity goals, and some demonstrated sound understanding of the underlying social, political and environmental determinants of ill-health and health inequity. However, others indicated shortcomings in such understanding. There were indications in some PHN documents of ‘victim blaming’ in statements suggesting that the ill-health of people experiencing social disadvantage was due to their knowledge or attitude deficits. Issues of ‘health literacy’ (a term almost synonymous with victim blaming [30]) were also relatively prominent in PHN documents, predominantly framed as a deficit of individuals requiring remediation on their part.

The ideological position that individuals are responsible for their health (victim blaming), that was apparent in a few PHNs, ignores the underlying social, cultural and economic factors that hinder behaviour change [4]. It also provides governments of such neoliberal persuasion with justification for abrogating responsibility to mitigate such factors through regulation, or fund medical services for consequent illness [31]. The ‘victim blaming’ rhetoric of some PHNs is concerning - if they genuinely do not understand the underlying determinants of health and their core beliefs are of individual responsibility, their potential for planning and developing upstream health promoting interventions will be limited.

Health promotion strategies that seek to drive individuals to improve their health behaviour can generate positive health outcomes in some individuals, particularly if employed alongside other strategies [29]. However, they work best in high socioeconomic groups, and less among people who experience disadvantage, so risk exacerbating health inequalities [29]. Where PHNs are developing and commissioning behaviour change activities, they should do so with great care, informed by evidence and community engagement to ensure that interventions are contextually appropriate and equity-sensitive, and complement broader strategies that address the underlying causes of ill-health.

### Improving upstream health equity action

We found several examples of PHNs acting on the social determinants of health, though there is scope for much more. Experience from the UK shows that upstream health promotion action can be achieved by meso-level, regional health organisations. Clinical Commissioning Groups (CCGs), as well as commissioning clinical services, also support early interventions to address social determinants of health inequalities and social exclusion, and work with local partners to promote health and wellbeing [32]. Some metropolitan ‘Core Cities’ CCGs in deprived urban areas work with partner organisations to focus on employment or promote physical activity through urban design and transport interventions [32]. Another UK example is a general practice-based social prescribing and community development scheme that addresses social isolation, and significantly reduced unplanned hospital admissions [33]. Limited collaboration with local government has been identified as hindering Australian PHCOs’ intersectoral action on upstream health determinants [34]. The importance of intersectoral action and collaboration to address health inequity through action on the social determinants has long been recognised [6, 35–37]. With appropriate planning autonomy from the federal government, and adequate timeframes and resourcing, PHNs could collaborate with local government and other partners to strengthen, and advocate for collaborative upstream health equity action.

PHNs could also do more in terms of local leadership and broader advocacy to drive action on the social determinants of health, leveraging the power of local medical actors [38]. With their strong evidence of local health needs and inequities, they are well placed to advocate for targeted action to address priority upstream issues.

Much of the literature on improving PHC in Australia focusses on enhancing aspects of clinical care: accessibility, inter-disciplinary collaborative care, and reforming the fee-for-service funding structure to better enable chronic disease management and prevention [15, 39]. Our findings indicate opportunity to go further, in terms of embracing the comprehensive vision of PHC set out in the Alma Ata Declaration [6], reaching towards a system of PHC that as well as providing high quality, accessible, appropriate clinical healthcare services, also incorporates collaboration between sectors to act on the underlying social determinants of health and inequity. There are many lessons and examples from the Aboriginal community controlled health sector in Australia, of ways in which to integrate primary care clinical services and action on the social determinants of health for holistic, comprehensive, local PHC [40]. Learning from these examples more broadly, and directly collaborating with these organisations, could support PHNs to take actions or advocate on social determinants of health. Allowing PHNs greater autonomy, scope, time and funding to use their commissioning levers to implement elements of comprehensive PHC into ‘mainstream’ primary care service models will contribute to moving Australian PHC in the right direction for improving health equity.

### Improving the equity-orientation of planned activities

While we found some PHN activities addressed equity of access to and quality of PHC services, most planned activities did not. There was very little indication of PHNs employing systematic mechanisms to incorporate equity considerations into their planning. While rigorous, detailed planning may appear aspirational in meso-level PHC planning organisations, in Canada some similarly small regional health organisations have clear processes and simple tools to support equity-oriented health planning and program development [41–43]. Oxman et al. [44], have suggested a simple, four-question approach to guide considerations of health inequity impacts in policy development. A standard risk-assessment matrix including consideration of equity implications of the planned activity could be included in PHNs’ planning process, however we identified no such mechanisms. Even within their somewhat limited scope of action, there are simple procedural changes that PHNs could make to plan in a way that considers health inequities.

A further element of equity particularly relevant to First Nations populations is cultural safety. While our study identified many indications of intentions and activities to promote cultural safety of PHC services, there was no indication that PHNs used any kind of evidence-based framework, such as that developed by Mackean et al. [45], to develop or commission culturally safe activities.

### Evidence-informed equity-oriented planning

A key principle of equity-oriented policy and planning is that strategies should be based on appropriate research, monitoring and evaluation [36]. Evidence is vital to equity-oriented planning – collecting information about health inequities is fundamental to acting to address them [2, 17, 46]. Our findings about PHNs’ use of evidence (reported elsewhere [47]) have some important implications for the equity-orientation of their planning. While we found widespread use of evidence to identify variations in population health needs, the PHNs’ minimal use of intervention evidence of ‘what works’, let alone ‘what works for whom, and how’ hinders equity-oriented intervention planning. There appears to be little consideration of the equity impacts of planned initiatives. PHNs may be commissioning initiatives that have no impact on equity, or even exacerbate health inequity in their regions. Greater use of equity-sensitive intervention evidence to inform PHC planning could help to ensure PHN activities reduce, or at least don’t worsen, health inequities.

Our research has successfully employed Freeman and colleague’s [17] framework for assessing PHCO health equity actions, and demonstrated its utility for highlighting strengths and weaknesses. Our application indicates it could be applied for assessing the actions of organisations, to identify opportunities for enhancing action on health inequities at the local level, and fostering more equity-oriented health systems more broadly.

### Limitations

We acknowledge the limited scope of our document analysis which only examined ‘core, flexible funding’ activity work plans, but not other planning documents regarding mental health or health of First Nations people, that PHNs also produce. As such there may have been some equity actions that we did not identify. However, our approach was similar to that of Freeman et al. [17] so we are confident of the validity and comparability of the research. The comprehensive interviews we conducted at each case study PHN also offset the risk that we failed to identify any major equity initiatives.

Our examination of community participation may have been strengthened by more focussed analysis of PHN stakeholder engagement frameworks (if they exist), and reports and analyses of consultation activities. Nonetheless, our analysis reliably indicates a moderate degree of community participation.

We also acknowledge that the interview data is somewhat dated, having been collected in 2016 and 2018, however the large number of interviews is a strength that offsets this limitation.

## Conclusions

Regional PHC organisations, and similar decentralised health planning agencies, are well placed to address health equity in their regions through a range of actions. For them to do so will require their funding body to value fairness, and appreciate that action on the underlying social determinants of health, is equally important as clinical healthcare services. Together they form a holistic, comprehensive ‘version’ of primary health care that addresses inequity more comprehensively. It will also require political will to allow PHC organisations scope to act on the ‘upstream’ factors that contribute to local health issues [48]. Primary health care organisations would also require sufficient time and resources for robust, systematic, evidence-informed participatory planning processes. To optimise regional planning for health equity incorporating established equity-promoting actions, organisations should engage meaningfully with local communities and service providers, incorporate mechanisms into their planning processes to embed equity considerations, and use evidence to ensure approaches are equity sensitive and appropriately targeted.

## Data Availability

Stakeholder interview and PHN internal documents data are not available, as the participants did not give consent for this. PHN external documents (91) were obtained from PHN websites, copies of which can be provided by the corresponding author upon request.

## Abbreviations

CCG: Clinical Commissioning Group
ML: Medicare Local
PHC: Primary Health Care
PHCO: Primary Health Care Organisation
PHN: Primary Health Network

## Footnotes

1 Metro North and Metro South are used here as anonymising pseudonyms and should not be confused with the PHNs or health services in Brisbane, which frequently also go by these short-hand names.

## References

[1] Braveman P, Gruskin S (2003). Defining equity in health. J Epidemiol Community Health.

[2] Marmot M, Friel S, Bell R, Houweling TAJ, Taylor S (2008). Closing the gap in a generation: health equity through action on the social determinants of health. The Lancet.

[3] Wilkinson R, Pickett K (2010). The Spirit Level: why equality is better for everyone.

[4] Baum F. The New Public Health. 3rd ed. Oxford University Press; 2008.

[5] Public Health Information Development Unit (PHIDU) TUA. Social Health Atlas of Australia: Population Health Areas (online) 2023. Accessed 1. November 2023. [Available from: https://phidu.torrens.edu.au/current/maps/sha-aust/pha-double-map/aust/atlas.html.

[6] World Health Organization. Alma Ata Declaration. Geneva, 1978.

[7] Starfield B, Shi L, Macinko J (2005). Contribution of primary care to health systems and health. Milbank Q.

[8] Macinko J, Starfield B, Shi L (2003). The contribution of primary care systems to health outcomes within Organization for Economic Cooperation and Development (OECD) countries, 1970–1998. Health Serv Res.

[9] Galea S, Kruk ME (2019). Forty years after Alma-Ata: at the Intersection of Primary Care and Population Health. Milbank Q.

[10] Koo D, Felix K, Dankwa-Mullan I, Miller T, Waalen J. A call for action on primary care and public health integration. Am J Public Health. 2012;102(S3). 10.2105/AJPH.2012.300824. 10.2105/AJPH.2012.300824 PMC3478083 22690962

[11] Scutchfield FD, Michener JL, Thacker SB (2012). Are we there yet? Seizing the moment to integrate medicine and public health. Am J Public Health.

[12] Checkland K, Coleman A, McDermott I, Segar J, Miller R, Petsoulas C (2013). Primary care-led commissioning: applying lessons from the past to the early development of clinical commissioning groups in England. Br J Gen Pract.

[13] Martin-Misener R, Valaitis R, Wong ST, Macdonald M, Meagher-Stewart D, Kaczorowski J (2012). A scoping literature review of collaboration between primary care and public health. Prim Health Care Res Devevlopment.

[14] Thompson J. The costly abolition of Medicare Locals. Australian Policy Online [Internet]. 2015 12 May 2017. Available from: http://apo.org.au/node/58917. [Accessed 12 May 2017].

[15] Swerissen H, Duckett S, Grattan Institute. Chronic Failure in Primary Care. ; 2016 March. Available from: https://grattan.edu.au/wp-content/uploads/2016/03/936-chronic-failure-in-primary-care.pdf [Accessed 1 September 2023].

[16] Booth M, Hill G, Moore M, Dalla D, Moore M, Messenger A. The new Australian Primary Health networks: how will they integrate public health and primary care? Public Health Research & Practice. 2016;26(1). 10.17061/phrp2611603. 10.17061/phrp2611603 26863166

[17] Freeman T, Javanparast S, Baum F, Ziersch A, Mackean T (2018). A framework for regional primary health care to organise actions to address health inequities. Int J Public Health.

[18] Australian Bureau of Statistics. Remoteness Areas. Australian Statistical Geography Standard (ASGS) 2023 [updated 21/3/23; cited 2023 27 October]. Edition 3:[Available from: https://www.abs.gov.au/statistics/standards/australian-statistical-geography-standard-asgs-edition-3/jul2021-jun2026/remoteness-structure/remoteness-areas.

[19] Javanparast S, Freeman T, Baum F, Labonté R, Ziersch A, Mackean T (2018). How institutional forces, ideas and actors shaped population health planning in Australian regional primary health care organisations. BMC Public Health.

[20] Szabo V, Strang VR (1997). Secondary analysis of qualitative data. Adv Nurs Sci.

[21] QSR International Pty Ltd. NVivo (version 12). 2018.

[22] Department of Health. Primary Health networks: Grant Programme guidelines. Australian Government; 2016.

[23] Popay J, Whitehead M, Hunter DJ. Injustice is killing people on a large scale—but what is to be done about it? Oxford University Press; 2010. pp. 148–9. 10.1093/pubmed/fdq029. 10.1093/pubmed/fdq029 20423920

[24] Baum F, Ziersch A, Freeman T, Javanparast S, Mackean T (2020). Strife of interests: constraints on integrated and co-ordinated comprehensive PHC in Australia. Soc Sci Med.

[25] Holding E, Fairbrother H, Griffin N, Wistow J, Powell K, Summerbell C (2021). Exploring the local policy context for reducing health inequalities in children and young people: an in depth qualitative case study of one local authority in the North of England, UK. BMC Public Health.

[26] Hunter DJ, Popay J, Tannahill C, Whitehead M (2010). Getting to grips with health inequalities at last?. BMJ.

[27] Bournival V, Oostlander SA, O’Sullivan TL. Lifestyle drift’ in Disaster risk reduction practices magnifies inequities for high-risk populations. SSM - Qualitative Research in Health. 2022;2. 10.1016/j.ssmqr.2022.100190.

[28] Baum F (2011). From norm to Eric: avoiding lifestyle drift in Australian health policy. Aust N Z J Public Health.

[29] Baum F, Fisher M (2014). Why behavioural health promotion endures despite its failure to reduce health inequities. Sociol Health Illn.

[30] Jamrozik K (2010). Health literacy, victim blaming and the mission of public health. Aust N Z J Public Health.

[31] Crawford R (1977). You are dangerous to your health: the ideology and politics of victim blaming. Int J Health Serv.

[32] NHS Clinical Commissioners. Shaping healthy cities and economies: The role of clinical commissioning 2016. Available from: https://445oon4dhpii7gjvs2jih81q-wpengine.netdna-ssl.com/wp-content/uploads/2016/12/NHSCC-Core-Cities-2016-Final.pdf. [Accessed 24 July 2020].

[33] Abel J, Kingston H, Scally A, Hartnoll J, Hannam G, Thomson-Moore A (2018). Reducing emergency hospital admissions: a population health complex intervention of an enhanced model of primary care and compassionate communities. Br J Gen Pract.

[34] Javanparast S, Baum F, Freeman T, Ziersch A, Henderson J, Mackean T (2018). Collaborative population health planning between Australian primary health care organisations and local government: lost opportunity. Aust N Z J Public Health.

[35] Kickbusch I (2008). Policy Innovation for Health. Kickbusch I.

[36] Whitehead M (1991). The concepts and principles of equity and health. Health Promot Int.

[37] Baum F (2018). Governing for health.

[38] Baum FE, Bégin M, Houweling TA, Taylor S (2009). Changes not for the fainthearted: reorienting health care systems toward health equity through action on the social determinants of health. Am J Public Health.

[39] Mengistu TS, Khatri R, Erku D, Assefa Y (2023). Successes and challenges of primary health care in Australia: a scoping review and comparative analysis. J Global Health.

[40] Pearson O, Schwartzkopff K, Dawson A, Hagger C, Karagi A, Davy C (2020). Aboriginal community controlled health organisations address health equity through action on the social determinants of health of Aboriginal and Torres Strait Islander peoples in Australia. BMC Public Health.

[41] Fraser Health. Community Planning Tool: Applying a Health Equity Lens to Program Planning. Surrey (BC): Fraser Health. ; 2018. Available from: https://www.fraserhealth.ca/-/media/Project/FraserHealth/FraserHealth/Health-Topics/20180322_Community_Planning_Tool_Online.pdf [Accessed 1 September 2023].

[42] Guichard A, Ridde V, Nour K, Lafontaine G. Taking better account of social inequalities in health - the REFLEX-ISS tool. Longueuil: CISSS de la Montérégie-Centre, Direction de santé publique de la Montérégie; 2015. Available from: http://www.equitesante.org/chair-realisme/tools/reflex-iss/ [Accessed 1 September 2023].

[43] Pauly B, MacDonald M, Hancock T, O’Briain W, Martin W, Allan D et al. Health Equity Tools. Victoria, BC: University of Victoria; 2016. Available from: Available from www.uvic.ca/elph. [Accessed 1 September 2023].

[44] Oxman AD, Lavis JN, Lewin S, Fretheim A (2009). SUPPORT tools for evidence-informed health policymaking (STP) 10: taking equity into consideration when assessing the findings of a systematic review. Health Res Policy Syst.

[45] Mackean T, Fisher M, Friel S, Baum F (2019). A framework to assess cultural safety in Australian public policy. Health Promot Int.

[46] Andermann A, Pang T, Newton JN, Davis A, Panisset U (2016). Evidence for Health I: producing evidence for improving health and reducing inequities. Health Res Policy Syst.

[47] Windle A, Javanparast S, Freeman T, Lo K, Baum F. Use of evidence to inform regional primary health care planning. Manuscript submitted for publication. 2023.

[48] Baum F, Townsend B, Fisher M, Browne-Yung K, Freeman T, Ziersch A (2022). Creating political Will for Action on Health Equity: practical lessons for Public Health Policy actors. Int J Health Policy Manage.

